# Supplementary material for: Field assessment in Cameroon of a reader of POC-CCA lateral flow strips for the quantification of Schistosoma mansoni circulating cathodic antigen in urine
Source: PLoS Negl Trop Dis. 2021 Jul 14;15(7):e0009569. doi: 10.1371/journal.pntd.0009569 (PMC8312929; doi:10.1371/journal.pntd.0009569)
Supplement: Attachment — Submitted filename: New Answers to Comment reviewers.docx [file pntd.0009569.s001.docx]

**Answer to reviewers comments**

**Reviewer #1**

**Introduction**

- L90: Why (sub)?

Answer

"Sub" has been deleted and the sentence has been rephrased as: *"Schistosomiasis is one of the most common neglected tropical diseases (NTD) and remains a major public health problem in many tropical and subtropical countries"*

- L100: (SAC)

Answer

"SAC" has been inserted in brackets in front of "school aged children"

- L102: After introducing the abbreviation PZQ please continue to use it out not introduce it at all

Answer

The reviewer suggestion has been taken into account and the abbreviation of praziquantel has been used accordingly in the manuscript

- L104: Rather than 'the real' I suggest 'accurate'

Answer

"real" has been changed to "accurate" as suggested by the reviewer

- L106: You are not just monitoring prevalence but also 'intensity'. I suggest you use the term progress towards control.

Answer

This review suggestion has been taken into account and the sentence has been rewritten as: *"Determining the accurate disease prevalence and the infection intensity in different communities with reliable tools appears therefore as the keystone for monitoring progress towards control of schistosomiasis in endemic areas."*

- L110: I don’t agree it is especially low-cost as training and retaining capable microscopists is expensive.  You have not described the cost of POC-CCA or the LR3 reader for comparison.

Answer

Information regarding the costs related to performing a single KK and POC-CCA have been included in the manuscript as stated in the following sentence:

"*In children of western Kenya for instance, Worrell et al. (2015) have shown that US$ 6.89 are spent per-person for a single KK against US$7.26 for POC-CCA. Although these analyses took into account the costs linked to labor, transportation and supplies, they didn't consider other costs (susceptible to increase the KK cost) like those related to the training and retaining capable microscopists."*

- L112: How important are these low intensity infections in terms of control defined as <1% moderate and high intensity infections (MHI).  If an infection in undetectable by KK hasn’t ‘control’ has already been achieved?  Are you suggesting elimination is the gaol?

Answer

The low intensity of infection may have some impacts in term of the number of eggs release in the environment and consequently, on the disease transmission. However, it doesn’t mean that the control has been achieved because the disease prevalence can still be high. It for these reasons that the following sentence was written:

"*On the basis of KK data, the disease prevalence is generally underestimated, and this will have an impact on the implementation of PCT programmes****.****»*

- L120: Stool 'or urine'.

Answer

The word "stool" here is correct because the topic is related to intestinal schistosomiasis. To clarify this point, S. mansoni has been inserted in the sentence that has been rephrased as: *"CCA in the urine is evidence of the presence of adult worms even if S. mansoni eggs cannot be found in stool"*

- L131: As stated previously there appears to be 4 objectives, please deal with them separately in this last sentence.

Answer

This last sentence of the introduction has been rewritten as: *"To overcome these difficulties, this study was designed with the aim of diagnosing S. mansoni infections and accurately quantifying CCA and hence infection intensity on POC-CCA cassettes or urine samples using the ESEquant LR3 reader for the first time in the field and compare results from KK and POC-CCA as well as those generated by POC-CCA on fresh and frozen urine samples."*

- L135: The roman numbering is unnecessary in these headings, please delete.

Answer

It has been deleted

**Methods**

- L144: What about younger children?

Answer

For younger children, only the signed consent form from their parents was considered as stated in the study protocol submitted to the national ethical committee. The sentence has been rephrased as: *"In addition, children of 10 to 14 years signed an assent form while for younger ones, only the consent form signed by their parents or guardians was considered."*

- L146: In the study

Answer

It has been corrected, ‘to the study’ was changed by ‘in the study’

- L150: WHO recommendations

Answer

It has been changed as suggested

- L165: Has there been any Schistosomiasis intervention in this area?

Answer

Intervention on schistosomiasis is performed in this area through mass administration of PZQ. The following sentence has been added at the end of this paragraph: *"Mass administration of PZQ is yearly performed in this locality."*

- L166: Readers will be interested in the range of prevalence and intensity found previously

Answer

Some previous prevalence have been inserted in the following sentence: *"Schistosoma mansoni infections have been reported in villages of Makenene for more than 30 years with prevalence ranging from 82% in 1987 to 18% in 2001*"

- L169-172: Please provide information on how these schools and participants selected (convenience or random sampling)?

Answer

Information on how schools and participants have been selected are provided in the following sentence: *"These schools were randomly selected and in each of them, stool and urine samples were collected from all children who accepted to participate in the study."*

- L190 and 194: Stick with the abbreviation KK

Answer

It was done

- L222: L 220-223 A figure illustrating the LR3 reader would be appreciated.

Answer

It was done

- L234 and L235: Grammar check.

Answer

It was done and the sentence has been rewritten as: *"The quantification of CCA on POC-CCA cassettes using G-scores is an innovative and user-friendly scoring system that records the intensity of CCA as 0, “trace”, 1+, 2+ or 3+ depending of their correspondence with G-scores"*

- L271 and 276: What do you mean by age group. Table 3 is not showing age groups but ages.

Answer

The word "group" has been deleted

- L279-280: Of the 71 by KK.

Answer

It was done as suggested

- L285: CCA

Answer

It was done as suggested

L304: Prevalence and intensity of S. mansoni by.

Answer

It was done as suggested. The title of the table has been changes as: *"Prevalence and intensity of S. mansoni by KK and POC-CCA in boys and girls"*

- L308 Table 3: Consider presenting this as age groups 5<10 years and 10<15 years the stat might be more interesting and a detailed describtion of epidemiology was not one of the main objectives

Answer

We have chosen to present results by age because we want to see if there is any correlation between age and infection intensity. However, when children were grouped (less than 10 years and >10 years) as suggested by the reviewer, no significant difference was observed regarding infection rate. Moreover, it will not be possible to know if there is any increase of CCA with age

- Table 2 and 3 Title: ‘Prevalence and intensity of…..

Answer

The titles of these tables have been changed.

*Table 2: Prevalence and intensity of S. mansoni infections by KK and POC-CCA in boys and girls*

*Table 3: Prevalence and intensity of S. mansoni infectionsby KK and POC-CCA according to age*

- Since examination of prevalence and intensity by age and by sex was not a defined objective perhaps the results should be more simply described by age group in Table 3 as it detracts for the other objectives of evaluating the LR3 reader in comparison with KK

Answer

We agree with the reviewer for the fact the prevalence and intensity by age and by sex were not a defined objective. However, we found relevant to see if the quantity of CCA could vary according to age. As previously mentioned, we want to see if there is any correlation between age and infection intensity. These information could be missed if children were grouped by ages.

- L312: No need of 'the abundance'

Answer

It has been deleted from the sentence

- L326: CCA

Answer

It has been corrected as suggested

- L331: This classification is expressed differently in methods:  light (<100) moderate (100-400) and heavy (>400). Please concur with the WHO classification.

Answer

It has been corected as suggested

- Lines 344 to 362 should be greatly simplified as table 4 illustrates the findings.

Answer

This section has been rewritten completely

- *"Out of 55 negative frozen samples, 49 (89.1%) were scored as trace (fresh samples). For the 6 remaining, 4 (7.3%) were scored 1+, one (1.8%) 2+ and the last (1.8%) 3+. Comparing results generated on fresh and frozen urine samples, good and significant correlations were observed with the reader (Pearson rho=0.85, P < 0.0001) (Figure 5) as well as the visual scoring system (Pearson rho=0.84, P < 0.0001). The means of CCA on POC-CCA strips decrease when these samples were stored for one year at -20° C (P < 0.001, F=6.00). Between fresh and frozen urine samples for instance, the mean values of POC-CCA decrease from: i) 69.32mv to 54.89 mV for samples with "trace" score; ii) 173.23mV to 150.92mV for those scored 1+; iii) 307.93mV to 275.25mV for samples with 2+ and; iv) 471.73mV to 398.67mV for samples having 3+. "*

- L385: Please describe the study limitations

Answer

Some limitations of the study have been raised. For instance, it was mentioned in the discussion the:

*"For the present study, it is important to point out that the infection rate revealed by KK has been probably underestimated because only one KK slide was examined for each child.*

*- Although information regarding the activities performed by boys and girls has not been collected during the present study, it is likely that boys and girls are involved in the same risky activities since they swim, wash clothes and fetch water in the same places."*

- L388: Prevalence 'and intensity' or perhaps 'disease burden'

Answer

The sentence has been rephrased as: *"These results confirm the low sensitivity of the KK test for the diagnosis of intestinal schistosomiasis in low disease prevalence and intensity settings"*

- L392-394: Could you please rephrase this sentence is not clear.

Answer

The sentence has been rephrased as: *"In such context, some children and especially those with low infection intensity of S. mansoni could pass undetected when only one KK slide is screened. This has been already demonstrated in previously studies"*

- L403: It would be nice to give possible reasons for these risky activities. What is the situation like in Makenene?

Answer

The possible reasons have been given in the following sentence: *"Although information regarding the activities performed by boys and girls has not been collected during the present study, it is likely that boys and girls are involved in the same risky activities since they swim, wash clothes and fetch water in the same places."*

- L410: What do you mean by 'younger' pre-SAC or the age group 5<10?

Answer

In this context, "younger children" refers to children of 5 to 9 year. The sentence has been rephrased as: "*In Makenene for instance, children of 5 to 9 years follow their elders in different risky activities such as swimming, washing clothes and fetching water".*

- L412: SAC

Answer

It was corrected as suggested

- L415: No need for 'or worm burden'

Answer

It was corrected as suggested

- L416: Rather than 'previous authors it is easier for the reader if you say 'where'

Answer

It was corrected and the sentence has been rewritten as: *"These results are in agreement with those obtained in Yemen and Cameroon"*

- L417: The discussion on age and sex could be more concise as the 'participation in risky behavior' is repetitious.

Answer

This part of the discussion has been rewritten as followed: *"They could be explained, in part, by the fact that boys were found to be swimming regularly in the river compared to girls."*

- L445: KK

Answer

It was corrected as suggested

- L447: You have made no mention of costs of the lR3 reader and until now no mention that it requires electricity which may be unavailable in many POC settings.

Answer

The cost of the reader is now mentioned in the sentence: *"However, electricity which may be unavailable in many settings and additional investment to purchase the reader that costs about 4233 euros are required compared to the visual scoring system".*

- L457-458: I know the cellophane has to be soaked for 24hrs to be used then slides can be read as long as the faecal smear is spread evenly on the slide.  Please clarify.

Answer

We agree with the clarification provided by the reviewer. The sentence has been rephrased as: *"In addition to the time spent to prepare and read the KK slides, the slides will be left for one or more hours at ambient temperature as preconized by the WHO for the clarification of the slides before they could be visualized under microscope."*

- L464: Does a second slide need to be taken from a second stool sample. Isn't another part of the same stool sample sufficient?

Answer

The second slide is usually performed on the same stool sample. The sentence has been rewritten as followed: "*In view of the requirement for skilled technicians for the KK test and the number of days required for each field survey, the survey costs to determine the prevalence and the intensity of S. mansoni infections will often be higher when using the KK, particularly if another KK slide is examined even if it is performed on the same stool sample"*

- L465: Do you mean intensity of infection? Doesn’t worm burden imply both prevalence and intensity combined?

Answer

In the sentence, "worm burden" refers to "intensity of infection". In consequence, "worm burden" has been replaced by "intensity of infection"

- L474: Is the WHO goal for SCH to eliminate or to control?  If it is to control then low levels of prevalence and intensity have already met that goal.

Answer

The WHO goal for schistosomiasis is to control. With the KK, the disease prevalence as well as the intensity of infections are underestimated and consequently, it is difficult to say that the control of schistosomiasis has been achieved. In the sentence, the term "eliminating" has been changed to "controlling".

- L480: Why use schistosome here rather than S. mansoni?

Answer

"schistosome" has been replaced by "*S. mansoni*"

- L484: Delete 'seems to'

Answer

It was done as suggested

- L492: Again I am sure you can find a way of expressing this in a more concise clear manner in both results and here in discussion.

Answer

The sentence of this line has been rewritten as: *"The good correlations reported between data generated by the reader (Pearson coefficient =0.85) and those from visual scoring system (Pearson coefficient = 0.84) on fresh and frozen samples demonstrates the capacity of POC-CCA to reproduce similar results on samples stored at -20^0^ C for one year."*

- L503: Again simplify the results and this discussion of results as it is overly complicated

Answer

The sentence of this line has been rephrased as: *"The fact that 89% of negative frozen samples were scored as "trace" (low quantity of CCA) on fresh urine samples raises the following questions"*

**Conclusion**

- L 514: The whole paper need to be reorganized along the 4 (possible 5 if you include epidemiological description by age and sex) objectives and then the conclusion should follow this same sequence of organization and simplified interpretation if SCH control is defined as <1% MHI.

Answer

The conclusion has been rewritten and different objectives have been taken into consideration

**Abstract**

- L39: There seems to be at least 4 objectives in this study 1) compare prevalence by CCA with KK, 2) compare visual appraisal of CCA with the LR3 reader 2) compare fresh and frozen results and 4) compare intensity of infection by LR3 reader with KK.  Please clarify this in the abstract and make adjustments to the background, methods, methods, results and conclusion.

Answer

The objective of the study has been reformulated as: *"This study was designed to compare the prevalence and intensity of S. mansoni by KK and POC-CCA and quantify, on fresh and frozen urine samples, CCA using the visual scores and the ESEquant LR3 reader."*

- L 44: Describe the LR3 reader as a means of quantification of the degree of positively

Answer

This part of the methodology has been rewritten as: *"The prevalence and intensity of S. mansoni were determined using KK and POC-CCA. The degree of the positivity of of POC-CCA was estimated by quantifying CCA on fresh and frozen urine samples using visual scores and strip reader. The prevalence, the infection intensity as well the relative amounts of CCA were compared."*

- L48: Visual scoring of intensity by KK (epg) or (ep10ml?) versus visual scoring of POC-CCA. please clarify

Answer

As this study concerns intestinal schistosomiasis, the intensity of infection by KK is in epg

It has been mentioned in brackets

- L51: pleased define frozen (<-20C)

Answer

It was defined by putting into brackets <-20^0^C where frozen has been used for the first time.

- L58: what about the last objective comparing LR3 reader and KK for intensity?

Answer

Conclusion related to the objective has been reported in the following sentence: "*This study confirmed the low sensitivity of KK and the high capacity of POC-CCA to provide reliable data on the prevalence and intensity of S. mansoni infections.*"

**Author Summary**

- L65: Spell POC-CCA in full for the first time of use

Answer

This was done

- L66: Now you can use CCA

Answer

This was done

- L68-70 there were at least 4 objectives to this study. Best to clarify that from the start and deal with each separately as you proceed

Answer

The objective has been rewritten as: *"This study was designed with the aim of comparing the prevalence and intensity of S. mansoni infections by KK and POC-CCA and quantifying under field conditions on fresh and frozen (<-20^0^C) urine samples, CCA using the visual scores and the ESEquant LR3 reader"*

- L72: SAC defined by 5-15 years?

Answer

This was done as suggested

- L77: Better to define frozen at -20C earlier in this sentence and in the full abstract.

Answer

This was done

- Overall, I wonder what the paper is trying to convey.  Are you proposing the use of the LR3 reader as a substitute for KK (microscopy)?  If so, please provide more details about costs and the need for electricity.

Answer

The points raise by the reviewer have been taken into account in the following sentence: *"However, the lack of electricity in many schistosomiasis endemic areas and additional investment to purchase the reader that costs about 4233 euros are required compared to the visual scoring system."*

- Why spend so much attention on fresh v frozen samples? If the LR3 reader were to be used as a substitute for KK it would be used as a POC tool? The degradation of accuracy with freeze/thaw is to be expected but I don’t think it warrants the extensive point by point description in the results and discussion which need to be made more concise.

Answer

We believe that in large scale epidemiological studies, it will be possible to collect a lot of urine samples and store them before the analyses. This could occur in epidemiological settings where the population is important. In addition, instead of using several people to collect urine samples, the number of individuals in the collected team could be reduced by freezing samples before the analyses. These are the reasons why efforts have been concentrated on fresh versus frozen samples.

The following part of the discussion has been rewritten as: *"Although some of these negative frozen samples, especially those with "trace" scores on fresh samples might not have had schistosomiasis, it is most likely that these changes in results are due to degradation of CCA in storage. This could occur in remote areas of most developing countries where light shortage is common and the freezers can experience power outages leading several freeze-thaw cycles of samples.*"

**Reviewer #2:**

- Page 13, line 143-144: author mentioned that parents or guardians signing consent on behalf of the children and also mention some set of children signing an assent form, this is a contradicting statement.

Answer

We do not agree with the reviewer because following the recommendations of the ethical committee, an assent form must be signed by children of 10 up to 18 years because they able to read and understand the information sheet. However, they don't have legally responsibility. For these children, the consent form signed by the parents or guardians as well as the assent form are requested. For others, only the consent form signed by parents or guardians are requested.

- Page 14, line 168-172: author did not state how the sample size was calculated and determine.

Answer

The formula to estimate the sample size has been included

- Page 15, line 188-202: KK examination was carried out on fresh stool samples just once, subsequence examination should have been employed there is disparity in the number of eggs per gram in the stool samples.

Answer

We agree with the reviewer. This has been taken into consideration in the discussion as stipulated in the following sentence: *"For the present study, it is important to point out that the infection rate revealed by KK has been probably underestimated because only one KK slide was examined for each child. In such context, some children and especially those with low infection intensity of S. mansoni could pass undetected when only one KK slide is screened. This has been already demonstrated in previously studies*"

- Page 14, line 169- 172: numbers of samples collected at each school were not stated.

Answer

The number of sample collected in each school has been inserted in the result section entitle field survey as follows: “*Amongst these children 387 were from the Bilingual primary school of Baloua, 208 from the public school of Makenene and 164 from the public school of Ngokop.”*

- Page 16, line 228: The specific type of drawer where POC-CCA cassette was removed wasn’t well stated, unlike line 222, which state each cassette was introduced into the drawer of the ESEQuant

Answer

Specifications on the drawer as well as the reader have included in the manuscript in the following sentences: *"For the visual scoring system, each POC-CCA cassette was removed from the drawer (Figure 1; Qiagen, Reference number ESLR11-MA-1140) of the ESEquant LR3 reader (Figure 1) and the intensity of CCA was scored by another person using G-scores as described by Casacurberta et al.* *[41]*". In addition, a picture or the reader showing the drawer has been included in the manuscript

- Page 16, line 229:” G-scores as described by”…not well stated.

Answer

This was done. The refence *"Casacurberta et al. [41]"* was added

**Reviewer #3:**

- It appears that there are prior studies that have used either KK or CCA to estimate prevalence in these communities. Can the authors state those prior estimates to give the reader and initial understanding about the appropriateness of sample size?

Answer

The point raises the reviewer has been considered and part of the discussion has been rewritten as followed: "*The S. mansoni infection rate of 40.7% inferred by POC-CCA is lower than 69.8% obtained 8 years ago in this same locality [28]. Regarding the infection rates by KK, our value of 9.4% is lower than 36.26% and 49% reported 7 and 25 years ago [37, 43]. This reduction is in agreement with what is observed in most schistosomiasis endemic regions and could be explained by the mass administration of PZQ initiated in schistosomiasis endemic areas of Cameroon since 2007 [43].*'

- The analysis is clearly presented and aligns well with the analysis plan stated. A valuable addition to the paper would be a visual comparison of the CCA cassettes that were classified as 0, “trace”, 1+, 2+ or 3+. Interpretation of the results would be augmented by an understanding if mass drug administration of praziquantal has been ongoing in this sample prior to the study.

Answer

The point raises the reviewer has been taken into account by adding the following sentence: *"Indeed, following annual mass distribution of PZQ to SAC, a reduction of schistosomiasis prevalence from 81.60% to 41% has been reported from 1985 to 2010 [43]."*

- On line 417 of the Discussion it states "In the present study, boys were found to swim regularly in the river." It is not clear how this observation was made. How were the data collected? If by questionnaire please add a table to describe these behaviors. If anecdotal please state it as such rather than as part of this study.

Answer

This part of the discussion has been rewritten and some sentences have been rephrased as: "*They could be explained, in part, by the fact that during the field surveys, boys were observed to be swimming regularly in the river compared to girls."*

*-* In Reference to the discussion of keeping the urine samples frozen at -20C on Line 485, can the authors discuss the potential for antigen degradation in the frozen urine samples if the freezers experienced power outages or other technical issues? It will be helpful to know if the samples analyzed when through freeze-thaw cycles.

Answer

This part of the discussion has been rewritten and the following sentence has been added in the discussion: *"This could occur in remote areas of most developing countries where light shortage is common and the freezers can experience power outages leading several freeze-thaw cycles of samples."*

- Prior understanding of MDA exposure in this population will help readers to interpret the age-stratified intensities presented in this work.

Answer

The following sentences have been added to show that MDA is annually performed in this region.

*"This reduction is in agreement with what is observed in most schistosomiasis endemic regions and could be explained by the t mass administration of PZQ initiated in schistosomiasis endemic areas of Cameroon since 2007 [43]. Indeed, following annual mass distribution of PZQ to SAC, a reduction of schistosomiasis prevalence from 81.60% to 41% has been reported from 1985 to 2010 [43*]."
